# Muscle Quality and Functional and Conventional Ratios of Trunk Strength in Young Healthy Subjects: A Pilot Study

**DOI:** 10.3390/ijerph191912673

**Published:** 2022-10-03

**Authors:** Waleska Reyes-Ferrada, Ángela Rodríguez-Perea, Luis Chirosa-Ríos, Darío Martínez-García, Daniel Jerez-Mayorga

**Affiliations:** 1 Department Physical Education and Sports, Faculty of Sport Sciences, University of Granada, 18071 Granada, Spain; 2 Exercise and Rehabilitation Sciences Institute, School of Physical Therapy, Faculty of Rehabilitation Sciences, Universidad Andres Bello, Santiago 7591538, Chile

**Keywords:** isokinetic, core muscles, antagonist/agonist, muscle strength, dynamometer

## Abstract

Background: The trunk strength conventional ratio (CR) has been evaluated. However, the functional ratio and the ratio of strength to body weight (BW) or muscle mass (MM) have been poorly explored. Relative strength is a measure of muscle quality. Objectives: To analyze the trunk strength ratio normalized by BW and MM and compare the trunk’s conventional and functional ratios collected in isokinetic and isometric conditions. Methods: Twenty-seven healthy males (21.48 ± 2.08 years, 70.22 ± 7.65 kg) were evaluated for trunk isometric and isokinetic strength using a functional electromechanical dynamometer. Results: The extensor’s strength was greater than the flexors, with a CR of 0.41 ± 0.10 to 0.44 ± 0.10. Muscle quality was higher in eccentric contraction and high velocity for flexors and extensors. The functional flexor ratio (FFR) ranged between 0.41 ± 0.09 and 0.92 ± 0.27. The functional extensor ratio (FER) ranged between 2.53 ± 0.65 and 4.92 ± 1.26. The FFR and FER showed significant differences between velocities when considering the peak strength (*p* = 0.001) and mean strength (*p* = 0.001). Conclusions: Trunk extensors were stronger than the flexors; thus, the CR was less than one. Muscle quality was higher at a high velocity. Unlike CR, FFR and FER behaved differently at distinct velocities. This finding highlights the need to explore the behavior of the functional ratio in different populations.

## 1. Introduction

The spine and the muscles surrounding it play an essential mechanical role in human function [1]. The neuromuscular system is crucial for maintaining the spine’s mechanical stability, increasing its ability to generate tension to avoid injuries [2]. Thus, muscles are the only voluntary support of the joints and act to initiate or accelerate movement and limit or decelerate movement [3]. In this way, agonist and antagonist musculature play an essential role in movement control [4].

It has been shown that a particular ratio exists between the agonist and the antagonist’s muscles to protect the joint [5]. An imbalance between antagonist/agonist (A/A) could mean a deficiency in the antagonist’s muscle to produce enough strength to slow down the agonist’s action in a movement, increasing the probability of ligamentous or muscle injury [6]. The conventional ratio A/A has been evaluated in certain joints and with different methods [7,8]. Concerning the trunk, studies in healthy subjects reported a conventional flexor/extensor’s ratio (F/E) of 0.84 (0.54–1.16), i.e., the isokinetic strength values of trunk extensors are greater than the flexors [9]. This ratio has been classically evaluated using an isokinetic dynamometer and calculated using peak torque [9,10], which has the limitation of not characterizing the inherent torque variation to the length–tension relationship [10]. Moreover, the conventional ratio does not consider the eccentric muscular action of the antagonist.

Trunk musculature depends on the context [11,12]; thus, the conventional ratio may not adequately explain the dynamics of movement in activities of daily living or sports. The functional ratio represents the eccentric antagonist/concentric agonist actions [13]. Functionally, the trunk muscles interact in an eccentric/concentric mode [14]. For example, the bending forward movement, commonly identified as a risk factor for lower-back pain [15], depends on eccentric control of the extensor muscles for its execution [3,16]. Thus, knowing the trunk’s functional F/E and E/F ratio would allow us to understand its muscular dynamics. 

In this sense, another approach to complement the evaluation of muscle function is by estimating other ratios, such as relative strength [17]. Relative strength is considered a measure of muscle quality [18]. Classically, relative strength has been expressed as the ratio of strength to total body weight [19,20,21]; however, the strength per muscle mass ratio has been poorly explored. Assessing muscle quality can help identify individuals who might benefit from interventions that improve muscle quality and prevent functional deterioration [22]. Considering the importance of muscle strength balance for joint stability and injury prevention, in contrast with the limbs that can be evaluated by comparing them with the contralateral, the trunk does not have this possibility, ratios thus become more relevant. Therefore, this study aimed to analyze the ratio normalized by body weight and normalized by muscular mass in trunk strength, and to compare the trunk’s conventional and functional ratios using different strength variables collected in isokinetic and isometric conditions.

## 2. Materials and Methods

### 2.1. Participants

Twenty-seven physically active male student volunteers (age = 21.48 ± 2.08 years, body mass = 70.22 ± 7.65 kg, height = 1.75 ± 0.72 m, muscle mass = 32.57 ± 4.33 kg, and body mass index (BMI) = 22.99 ± 1.44 (kg/m^2^) without any experience in isokinetic or dynamometers devices were recruited from the university community. Participants were eligible for the study if they were (I) free from lower-back pain, with a maximum of 20% in the Oswestry Disability Index, and (II) free from musculoskeletal injury, especially health conditions affecting the spine, such as scoliosis, radiculopathy, and lower-back pain. All participants were informed regarding the nature, aims, and risks associated with the experimental procedure before giving their written consent to participate. The study protocol was approved by the Institutional Review Board of the University of Granada (n° 350/CEIH/2017) and by the Declaration of Helsinki.

### 2.2. Anthropometrics Measurements

Anthropometric measurements were performed following the guidelines of the International Society for the Advancement in Kinanthropometry (ISAK) [23], with the subjects being barefoot and wearing their underwear. Measurements were taken during the morning and prior to the strength assessment. All of the assessments were performed twice by an ISAK level 2 certified anthropometrist (W.R.-F.). Measurements included baseline measurements (weight, and standing and sitting height), ten perimeters (head, relaxed arm, flexed arm in tension, forearm, midsternal thorax, waist, hip, maximum thigh, mid-thigh, and calf), six skinfolds (triceps, subscapular, suprascapular, abdominal, front thigh, and medial leg), and six bone diameters (biacromial, transverse thorax, anteroposterior thorax, biiliocrestal, humeral, and femoral). Height (cm) was measured using a SECA stadiometer (SECA, Hamburg, Germany) with 0.1 mm precision, body weight (kg) using a SECA scale (SECA, Hamburg, Germany) with 100 g precision, and skinfolds with a Harpenden caliper (Harpenden, London, UK) with a precision of 0.2 mm. The rest of the measurements were performed with the Rosscraft anthropometric kit. Body composition was determined through Kerr’s equation using a Microsoft Office Excel spreadsheet [24].

### 2.3. Procedures

Subjects first attended a familiarization session. All of the evaluations were conducted by sports science researchers (Á.R.-P.) who had extensive experience performing muscle strength testing and using the device. Participants completed three isokinetic velocities (0.15 m·s^−1^ (V_1_), 0.30 m·s^−1^ (V_2_), and 0.45 m·s^−1^ (V_3_)), isometric conditions, and two range of movement (R) protocols. R was established by measuring the distance between the acromion and greater trochanter [25,26]. The order of the V and R was randomly established. Isometric (0 m·s^−1^, 90° between the trunk and thigh) and isokinetic strength were evaluated with a functional electromechanical dynamometer (FEMD) (Model Dynasystem, Granada, Spain) [27]. The reliability of the FEMD for trunk strength assessment in healthy subjects has been previously established [25,26].

### 2.4. Test Protocol

**Figure 1:**
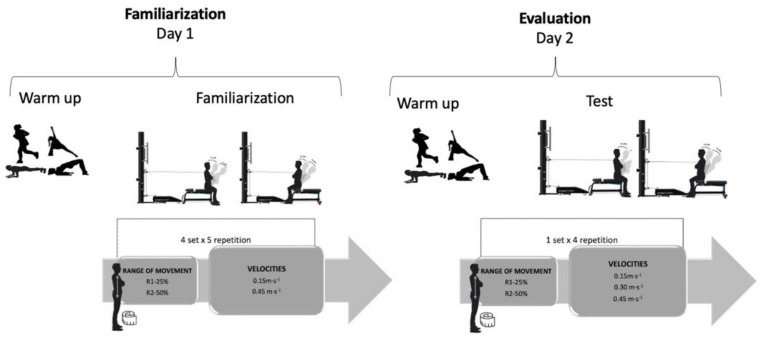
Protocol measurement of the trunk strength.

The participants were positioned in front of the FEMD to assess the extensor trunk strength and behind the FEMD to evaluate the flexor trunk strength. Each participant sat with their trunk at 90° on a bench, arms crossed on their chest, knees in flexion, and feet on the floor (Figure 1). Participants were then stabilized in the test position using straps. Sliding forward on the bench was avoided by using appropriate belts that pushed the pelvis and the legs down and back, but that were not uncomfortable for the participants.

Participants first attended a familiarization session with the FEMD, and a researcher explained the evaluation procedures. The familiarization consisted of a general warm-up that included 5 min of jogging, 5 min of joint mobility, and three sets of 15 s of frontal planks and glute bridges. The general warm-up was followed by four sets of five repetitions (two submaximal repetitions and three maximum repetitions) at V_1_ and V_3_ with R_1_ and R_2_, at 25% and 50% of R, respectively. The rest between sets was 3 min.

For the evaluation, familiarization and warm-up were performed, followed by 5 min of rest. The test consisted of one set of four maximum consecutive repetitions of trunk flexors and extensors at V_1_R_1_ (0.15 m·s^−1^, 25% cm), V_2_R_1_ (0.30 m·s^−1^, 25% cm), V_3_R_1_ (0.45 m·s^−1^, 25% cm), V_1_R_2_ (0.15 m·s^−1^, 50% cm), V_2_R_2_ (0.30 m·s^−1^, 50% cm), and V_3_R_2_ (0.45 m·s^−1^, 50% cm). The rest between sets was 3 min. After that, a maximum isometric contraction (0 m·s^−1^, 90 degrees) of 5 s in a seated position (formed 90 degrees between the trunk and thigh) was performed.

### 2.5. Muscle Quality and Trunk Ratios Strength

Muscle quality was measured through relative strength, i.e., trunk strength normalized by total body weight and trunk strength normalized by lean body mass. The ratios, peak, and mean strength were calculated using the average of both ranges of movement for each velocity. The three highest mean and peak strength repetitions for the concentric and eccentric contractions were taken to calculate the dynamic strength. Calculating the isometric strength, the repetition’s peak and mean values were taken. The A/A ratios were calculated for each velocity using both the peak and mean strength, with the following equations [13]:

Conventional ratio (CR) = (trunk flexors concentric strength)/(trunk extensor concentric strength)

Functional flexor ratio (FFR) = (trunk flexors eccentric strength)/(trunk extensor concentric strength)

Functional extensor ratio (FER) = (trunk extensor eccentric strength)/(trunk flexor concentric strength).

### 2.6. Statistical Analysis

Descriptive data were presented as mean ± standard deviation (SD). The Shapiro–Wilk normality test verified the normal distribution of the data. *T*-tests of the paired samples assessed the difference between ratios with the Cohen’s d effect size. The following scale was used for interpreting the magnitude of the effect size (ES): <0.20 = trivial, 0.20–0.59 = small, 0.60–1.19 = moderate, 1.20–2.00 = large, and >2.00 = very large [28]. A repeated-measures analysis of variance (ANOVA) was conducted with Bonferroni post-hoc analyses. The Greenhouse–Geisser correction was used when the Mauchly sphericity test was violated. Omega square (ω^2^) was calculated for the ANOVA where values of the effect size 0.01, 0.06, and above 0.14 were considered small, medium, and large, respectively [29]. A JASP software package (version 0.14.1, http://www.jasp-stats.org (accessed on 20 May 2022)) was used for all of the analyses. Statistical significance was set at *p* ≤ 0.05.

## 3. Results

The trunk extensors presented higher mean and peak strength values than the trunk flexors in the different conditions evaluated in the isometric and isokinetic testing. The highest strength value was recorded in the highest velocity in the eccentric contraction. When considering the peak strength or mean strength, the ratio normalized by body weight and the ratio by muscle mass was higher in V_3_ for both the extensors and flexors (Table 1).

There were no significant differences between the mean and peak strength of the conventional trunk strength ratio (*p* > 0.05) in isometric and isokinetic testing, except in the V_2_ condition (*p* = 0.02). On the other hand, both the functional flexor ratio and the functional extensor ratio had significant differences when considering the peak or mean strength in isokinetic conditions (*p* = 0.001), but not a significant difference in isometric conditions (*p* > 0.05) (Table 2).

No significant differences existed between the conventional ratio at the different velocities using peak strength (*p* = 0.145) or mean strength (*p* = 0.832). The conventional ratio was similar in all conditions; the highest functional flexor ratios were observed in V_3_ for peak strength (0.72) and mean strength (0.92), and the highest functional extensor ratios in V_2_ for peak strength (3.55) and V_3_ for mean strength (4.92) (Table 2).

For the functional flexor ratio, there were significant differences with a large effect size between velocities when considering the peak strength (*p* = 0.001; ω^2^ = 0.436) and mean strength (*p* = 0.001; ω^2^ = 0.447). The post hoc analysis using Bonferroni’s correction revealed significant differences between all three velocities compared with isometric conditions (*p* = 0.001), and between the ratio at V_1_–V_2_ (mean difference = −0.058; *p* = 0.009) and V_1_–V_3_ (mean difference = −0.099; *p* = 0.001) for peak strength. When considering the mean strength, the post hoc analysis revealed significant differences between all three velocities compared with the isometric conditions (*p* = 0.001), and between the ratio at V_1_–V_3_ (mean difference = −0.139; *p* = 0.001) and V_2_–V_3_ (mean difference = −0.087; *p* = 0.047) (Table 2).

For the functional extensor ratio, there were significant differences with a large effect size (*p* = 0.001; ω^2^ = 0.292) between velocities when considering the peak strength (*p* = 0.001; ω^2^ = 0.292) and mean strength (*p* = 0.001; ω^2^ = 0.461). The post hoc analysis revealed significant differences between all three velocities compared with the isometric conditions when considering the peak (*p* = 0.001) and mean (*p* = 0.001) strength (Table 2).

## 4. Discussion

The study’s purpose was to analyze the ratio normalized by body weight and normalized by muscular mass in trunk strength, and to compare the trunk’s conventional and functional ratios using different strength variables collected in isokinetic and isometric conditions. The main findings of this study were that (1) in all of the conditions studied, the strength values of the trunk extensors were higher than for the flexors; the highest strength value was recorded at the highest velocity in eccentric contraction. Thus, the strength per total weight or strength per muscle mass ratio was higher in V_3_. (2) There were no significant differences between the mean and peak manifestation of the conventional ratio, except for V_2_. (3) The functional ratios behaved differently when considering the peak or mean strength at all velocities, except in the isometric condition, but the conventional ratio did not differ between the three velocities or isometric strength. These findings suggest that the conventional and functional strength ratios behaved differently in the healthy subjects’ mean and peak manifestations.

Consistent with previous studies [9,21,30,31], the conventional ratio was below 1. The evidence demonstrates that the extensor group was stronger than the flexor group [9,31,32], which is consistent with this study’s findings. The highest absolute trunk strength was found in the flexor and extensor’s eccentric strength at the highest velocity, which explains why the highest relative strength was obtained in V_3_. Thus, according to the results, healthy subjects had an eccentric extensor strength of about twice the body weight, while for the flexors, it was about once the body weight when the peak strength was considered. This data, despite being derived from a small sample, could be helpful in sports and clinical settings, and an easy parameter to calculate. However, additional research is required in this area in order to identify the reference values.

The functional ratio has been less explored in the literature concerning the trunk. Considering the importance for joint stability, the eccentric to concentric relationship between A/A has been examined in the shoulder, as a representation of the cocking and spiking phases in volleyball players [7], and in the knee, in order to understand the importance of eccentric hamstring strength concerning quadriceps concentric strength in the prevention of anterior cruciate ligament injuries [8]. Considering the dynamic nature of the movements, perhaps exploring this ratio is of greater importance for understanding the functioning of the trunk musculature. Classically, the functional ratio accounts for the eccentric strength of the flexors and the concentric strength of the extensors (functional flexor ratio) [13], considering that the latter is responsible for initiating trunk extension from the upright position, which must be controlled eccentrically by the abdominals [16]. In this study, the functional flexor ratio ranged between 0.62 and 0.72 when considering the peak strength, and 0.78 and 0.92 when considering the mean strength. A novelty of this study is the incorporation of the functional extensor’s ratio. Let us consider that the extensors must eccentrically control the bending forward in daily activities and sports [16]. Therefore, this parameter should be considered, especially when we know that the extensor muscles are weaker in subjects with lower-back pain [9]. According to our data on healthy subjects, the eccentric strength of the extensor is up to four times the concentric strength of the flexors. In the post hoc analysis, the functional flexion and extension ratio showed significant differences for all velocities (V_1_, V_2_, and V_3_) compared with the isometric conditions.

Comparing the conventional ratios between different research is difficult because of the various devices and parameters used (position, velocities, and range of motion) [14,30]. Moreover, some authors use strength as an absolute value [21], and others are normalized by body weight [14]. Furthermore, this study used a linear device, so we did not express the strength concerning a joint moment. On the other hand, comparing functional ratios is even more difficult, as different authors measure different ratios, such as flexor eccentric/flexor concentric (Fecc/Fcon) or extensor eccentric/extensor concentric (Eecc/Econ) ratios. For example, Müeller et al. [33] reported a Fecc/Fcon between 1.01 to 1.05 and Eecc/Econ between 1.35 to 1.24 at 30°/s in healthy adolescent athletes. On the other hand, Dervisevic et al. [30] reported a Fecc/Fcon between 1.12 at 30°/s and 1.22 at 60°/s, and Eecc/Econ between 1.18 at 30°/s and 1.39 at 60°/s in a population similar to this study, i.e., in healthy young men, using a classic isokinetic dynamometer. Understanding the behavior of the antagonist and agonist muscles, i.e., co-contraction during trunk movements, is a phenomenon that should be studied.

Finally, this study is not exempt from limitations. First, the sample was relatively small, and we only evaluated male students without back pain or injury, so our data cannot be extrapolated to the rest of the population. Second, as previously explained, using this new generation of dynamometers that provide linear values made it challenging to compare our results with other studies. Third, although the participants did not have scoliosis or any other type of diagnosed postural alteration, we did not perform a formal assessment process for postural alterations in the sagittal plane (radiographic or clinical as the plumb line). However, during the assessment, we used a seated posture to minimize the influence of lower extremity dysmetria on trunk function. For future studies, we suggest performing an adequate postural assessment before evaluating trunk strength. Furthermore, we suggest assessing these ratios in different populations, such as women, the elderly, and subjects with lower-back pain, to establish the ratio behavior and to determine if there are differences between these different populations. In addition, we can suggest evaluating the rest of the core muscles in order to understand their synergistic role in the kinetics chains, and using other methods of estimating body composition to quantify trunk muscle mass precisely.

## 5. Conclusions

In conclusion, trunk extensors are stronger than flexors muscles, resulting in a conventional ratio lower than one. The normalized ratio per body weight or muscle mass is higher when considering the eccentric strength and high velocity (V_3_). The conventional ratio behaves similarly when considering the peak or mean strength, but the functional ratio is different for all velocities, except in isometric conditions. This finding highlights the need to explore the behavior of the functional ratio in diverse populations.

## Figures and Tables

**Table 1:** The absolute and relative strength of the trunk flexor and extensor muscles at different velocities.

		Peak Strength (*n* = 27)			Mean Strength (*n* = 27)		
		Strength (kg)	Normalized by Weight	Normalized by Muscular Mass	Strength (kg)	Normalized by Weight	Normalized by Muscular Mass
Extensors	Concentric						
	0.15 m·s^−1^ (V_1_)	95.91 ± 19.70	1.37 ± 0.26	2.97 ± 0.64	63.38 ± 15.61	0.91 ± 0.22	1.96 ± 0.51
	0.30 m·s^−1^ (V_2_)	95.87 ± 20.57	1.37 ± 0.28	2.98 ± 0.69	62.41 ± 16.94	0.89 ± 0.23	1.94 ± 0.55
	0.45 m·s^−1^ (V_3_)	101.34 ± 21.66	1.45 ± 0.32	3.16 ± 0.78	60.25 ± 16.39	0.86 ± 0.24	1.88 ± 0.55
	Eccentric						
	0.15 m·s^−1^ (V_1_)	136.30 ± 27.19	1.95 ± 0.36	4.23 ± 0.91	112.41 ± 24.25	1.61 ± 0.33	3.49 ± 0.80
	0.30 m·s^−1^ (V_2_)	141.23 ± 25.35	2.02 ± 0.34	4.38 ± 0.84	114.03 ± 24.10	1.63 ± 0.31	3.53 ± 0.76
	0.45 m·s^−1^ (V_3_)	149.26 ± 24.78	2.14 ± 0.37	4.64 ± 0.91	116.06 ± 25.19	1.66 ± 0.37	3.61 ± 0.88
	Isometric	93.73 ± 21.86	1.34 ± 0.29	2.90 ± 0.68	76.03 ± 20.37	1.09 ± 0.27	2.35 ± 0.62
Flexors	Concentric						
	0.15 m·s^−1^ (V_1_)	39.40 ± 5.45	0.56 ± 0.84	1.22 ± 0.20	25.34 ± 4.40	0.36 ± 0.06	0.78 ± 0.13
	0.30 m·s^−1^ (V_2_)	40.31 ± 6.45	0.58 ± 0.09	1.25 ± 0.22	24.64 ± 4.89	0.35 ± 0.06	0.76 ± 0.14
	0.45 m·s^−1^ (V_3_)	43.25 ± 7.49	0.62 ± 0.11	1.34 ± 0.27	24.19 ± 4.68	0.34 ± 0.06	0.74 ± 0.13
	Eccentric						
	0.15 m·s^−1^ (V_1_)	57.74 ± 7.49	0.83 ± 0.89	1.79 ± 0.23	46.93 ± 6.98	0.67 ± 0.09	1.45 ± 0.22
	0.30 m·s^−1^ (V_2_)	62.81 ± 8.48	0.90 ± 0.11	1.95 ± 0.26	49.21 ± 7.52	0.70 ± 0.09	1.52 ± 0.20
	0.45 m·s^−1^ (V_3_)	70.27 ± 9.51	1.01 ± 0.14	2.18 ± 0.35	51.95 ± 8.35	0.74 ± 0.10	1.61 ± 0.25
	Isometric	37.00 ± 5.69	0.53 ± 0.06	1.14 ± 0.15	30.47 ± 5.64	0.43 ± 0.06	0.94 ± 0.13

**Table 2:** Behavior of trunk strength ratios at different velocities.

Ratio	V_1_ Mean (SD) (*n* = 27)	V_2_ Mean (SD) (*n* = 27)	V_3_ Mean (SD) (*n* = 27)	ISO Mean (SD) (*n* = 27)	Repeated Measures ANOVA
CR—Peak	0.42 (0.09)	0.44 (0.10)	0.44 (0.09)	0.41 (0.10)	F (2,60) = 1.94, *p* = 0.145 ω^2^ = 0.006
FFR—Peak	0.62 (0.12)	0.68 (0.15)	0.72 (0.16)	0.41 (0.09)	F (2,59) = 121.16, *p* = 0.001 ω^2^ = 0.436
FER—Peak	3.49 (0.71)	3.55 (0.66)	3.51 (0.66)	2.55 (0.55)	F (2,60) = 52.59, *p* = 0.001, ω^2^ = 0.292
CR—Mean	0.42 (0.12)	0.42 (0.11)	0.43 (0.13)	0.42 (0.11)	F (2,54) = 0.19, *p* = 0.832 ω^2^ = 0.000
FFR—Mean	0.78 (0.21)	0.83 (0.21)	0.92 (0.27)	0.42 (0.11)	F (2,64) = 92.21, *p* = 0.001, ω^2^ = 0.447
FER—Mean	4.51 (1.03)	4.73 (1.09)	4.92 (1.26)	2.53 (0.65)	F (2,53) = 103.34, *p* = 0.001, ω^2^ = 0.461

V_1_ = 0.15 m·s^−1^; V_2_ = 0.30 m·s^−1^; V_3_ = 0.45 m·s^−1^; ISO = isometric; SD = standard deviation; CR = conventional ratio; FFR = functional flexor ratio; FER = functional extensor ratio.

## Data Availability

Not applicable.
